# PrEP Navigator Perceptions of the Implementation of Injectable PrEP on HIV Prevention in Tennessee

**DOI:** 10.3390/ijerph22050662

**Published:** 2025-04-23

**Authors:** Cristian J. Chandler, David G. Schlundt, Chloe Dagostino, Kemberlee R. Bonnet, Ashley J. Sellers, Latrice C. Pichon, Leah R. Alexander

**Affiliations:** 1 Division of Infectious Diseases, Vanderbilt University Medical Center, Nashville, TN 37203, USA; 2 Department of Psychology, Vanderbilt University, Nashville, TN 37240, USA; david.schlundt@vanderbilt.edu (D.G.S.); cd2292@mynsu.nova.edu (C.D.); kemberlee.bonnet@vanderbilt.edu (K.R.B.); ashley.j.sellers@vanderbilt.edu (A.J.S.); 3 School of Public Health, University of Memphis, Memphis, TN 38152, USA; lcpichon@memphis.edu; 4 Department of Public Health Practice, Meharry Medical College, Nashville, TN 37208, USA; lalexander@mmc.edu

**Keywords:** HIV, pre-exposure prophylaxis, PrEP, Tennessee, long-acting injectable, CFIR, implementation

## Abstract

Tennessee is in the southern region of the United States and has not yet fully benefitted from HIV pre-exposure prophylaxis (PrEP). Relatively little research has focused on pivotal roles of PrEP navigators. This study examined PrEP navigator perceptions of implementing long-acting injectable (LAI) PrEP in Tennessee. Semi-structured interviews with state-funded navigators were audio-recorded, transcribed, and systematically coded using a hierarchical system. Coded transcripts were aggregated, sorted, and analyzed using an iterative inductive/deductive qualitative approach. Using the Consolidated Framework for Implementation Research (CFIR), institutional, individual, and modifying factors to initiating and transitioning to LAI PrEP were identified. Most navigators initially had limited training and experience with LAI PrEP. Navigators reported systemic barriers associated with accessibility to LAI PrEP such as health insurance, pharmaceutical policies, and cost policies. While navigators noted the continued support of the state health department, strategies for circumventing individual and structural barriers are needed for universally implementing injectable PrEP.

## 1. Introduction

Although the southern United States accounts for an estimated 38% of the US population, greater than 50% of HIV incidence cases occur in the south annually [1]. This regional disparity is associated with sexual and gender minorities, people who inject drugs (PWID), and those unstably or unhoused, particularly in communities of color [2,3,4]. From 2015 to 2019, Tennessee saw an increase of approximately four percent in new HIV infections [5].

Despite the safety of pre-exposure prophylaxis (PrEP), the US South has not realized the promise of HIV prevention using PrEP. Since approval for use in 2012, PrEP has been an important biomedical HIV prevention tool. Historically, there has been slow uptake of PrEP in populations most at risk for HIV—Black and Latinx men who have sex with men (MSM), transgender women, and cis-gender Black women [6,7,8,9,10,11]. Social and structural impediments to PrEP initiation and persistence have been identified, including costs of medication and laboratory costs associated with PrEP maintenance, structural racism in healthcare systems, stigma associated with PrEP use, and concerns with side effects [8,10,12,13,14]. For PWID, barriers to PrEP initiation include intrapersonal barriers (i.e., competing health concerns, low perceived risk for HIV, low PrEP awareness), intrapersonal barriers (i.e., stigma), and structural barriers (i.e., cost of the medication, limited capacity among clinics serving PWID, criminal justice system involvement) [2]. PrEP access has often been subject to a patchwork system of programs to assist with the cost of the PrEP medication and, in some instances, the ongoing laboratory and medical services to maintain PrEP availability. Some programs have been led by drug manufacturers, and others by federal or state-specific programs, as well as 340B clinics which used discount drug rebates to support labs and visits [15]. Such complexity often led to confusion, as programs may have had differing applications and eligibility. To increase PrEP uptake, several areas, including the state of Tennessee, initiated a corps of PrEP navigators to identify and support candidates for PrEP [16].

PrEP navigation includes a wide-ranging set of skills and tasks aimed at increasing PrEP initiation, adherence, and persistence among populations most impacted by HIV. Current PrEP navigation is patterned after patient navigation strategies used to support addressing other disparities in the healthcare system [17,18]. PrEP navigation has also been informed by linkage, reengagement, and retention strategies in HIV care (NLRR) programs demonstrating improved HIV continuum of care outcomes in the Care and Prevention in the United States (CAPUS) Demonstration Project [19]. PrEP navigators often receive specialized training including HIV testing and risk reduction counseling, HIV PrEP and post-exposure prophylaxis (PEP) counseling, motivational interviewing techniques, healthcare benefits communication, referrals for wrap-around services (e.g., housing, STI screening), cultural competency trainings, and ending the HIV epidemic best practices including case management skills [16,20]. PrEP navigators may also directly communicate with insurers and pharmacies and coordinate care across multiple domains which can vary by locality [21].

An analysis of PrEP use from Tennessee’s participation in the Center for Disease Control and Prevention’s (CDC) Project PrIDE (PrEP, Implementation, Data2Care and Evaluation) demonstration project noted that there was a local disparity in PrEP uptake. Black individuals were less likely to be linked than white and Hispanic counterparts in Tennessee [22]. Variations in PrEP acceptance and linkage also varied by area of the state. Acceptance among transgender populations, while similar to that among cisgender counterparts, did not translate into filled PrEP prescriptions, indicating the need to better assess the needs of the population [22]. Discussions with community health workers in Tennessee revealed barriers to PrEP uptake at all five levels of the social ecological model: individual, interpersonal, community, organizational, and policy [23]. An evaluation of the PrEP navigators in the PrIDE study found several facilitators to PrEP engagement such as creating rapport with clients and staff, accompanying clients to initial appointments, and being able to talk about first-hand experience using PrEP in addition to regular contact. Identified barriers to PrEP uptake included assessing PrEP readiness, tracking clients, and instances of stigma socially or within healthcare settings [16].

In December 2021, the US Food and Drug Administration (FDA) approved a long-acting injectable (LAI) PrEP formulation. Previously, the only approved versions of PrEP were oral daily pills of tenofovir disoproxyl fumarate/emtricitabine or (tenofovir alafenamide/emtricitabine), known as brand names Truvada^®^ and Descovy^®^ respectively. After successful trials in HPTN 083 and HPTN 084 [24], the US Food and Drug Administration approved an LAI (cabotegravir extended-release injectable suspension) known as Apretude^®^ in December 2021 [25]. This newly approved form of PrEP has been studied in terms of acceptability [26,27]. It is not known if this PrEP modality increases uptake which will depend on overcoming barriers identified during the initial introduction of PrEP such as cost, availability, access, stigma and concerns about side effects [28,29,30,31,32]. To maximize PrEP initiation and PrEP persistence, it is critical to understand strategies that were successful in supporting PrEP users, and how these may be used to increase PrEP use as new modalities emerge. PrEP navigators may be a viable strategy for addressing the racial and regional disparities in uptake by focusing on assisting clients in initiating and maintaining PrEP use despite individual, social, and structural barriers [16].

This study utilizes semi-structured interviews to describe the implementation of LAI PrEP in Tennessee by PrEP navigators supported by the state health department. By bridging the gap between prescribing healthcare providers and clients who are poised to benefit from PrEP use, navigator insights into how to integrate LAI PrEP and potential new PrEP products may prove vital in addressing the ongoing HIV disparities faced in southern states like Tennessee.

## 2. Materials and Methods

This study was a cross-sectional, qualitative interview study. All procedures were approved by the Institutional Review Board at Vanderbilt University Medical Center (Protocol #221480). To be eligible to participate, PrEP navigators must have been employed as a PrEP navigator for a minimum of six months, and must have been at least partially funded (i.e., salary) by the state health department. PrEP navigators who were not supported by the state department of health and/or whose tenure of navigation was less than six months were excluded from this study. Sampling of navigators included an-IRB approved letter of invitation for the study that was emailed to potential participants from the state health department. The sampling frame included all possible PrEP navigators who participated in quarterly meetings with the state health department. Qualifying PrEP navigators were sent an interest letter via email by the statewide PrEP coordination office. Potential participants were those who agreed to be interviewed; they then used a link in the interest letter to schedule an appointment to be interviewed. Once the appointment was confirmed, interviews were conducted via telephone between October 2022 and April 2023.

Seven interviews were conducted with HIV PrEP navigators by trained, masters-level interviewers from Vanderbilt University Qualitative Research Core (AS, CD). The interviewers did not have contact with study participants prior to scheduling interviews. To ensure the trustworthiness of the qualitative data, interviewers were not part of the core research team, but rather part of the Qualitative Research Core, which is a service offered to the Vanderbilt research community. This separation minimized the potential for bias. By maintaining this independence, we aimed to gather more neutral and objective perspectives from participants, which contributed to the reliability of the data. Interview duration ranged from 16 to 58 min (median 34 min). A semi-structured interview guide was developed that included questions pertaining to (1) their role as a PrEP navigator; (2) their training experiences for providing LAI PrEP; (3) experiences initiating or transitioning clients to LAI PrEP; and (4) identifying needs in training navigators for LAI PrEP initiation or transition for clients. Follow-up questions were asked for clarity purposes and to facilitate detailed discussion. The guide was reviewed and revised iteratively with input from representatives at the state health department until the research team agreed that it would address our research questions. Questions included for this analysis are included in Appendix A.

All interviews were conducted online at a time chosen by the participants. Interviews were audio recorded. Audio files were submitted to an IRB-approved transcription service (rev.com) and transcribed verbatim. Study personnel maintained a recording log on a password protected server, housed in a locked office at Vanderbilt University. Transcripts were de-identified and each participant was assigned a unique participant ID number.

Qualitative data coding and analysis was managed by the Vanderbilt University Qualitative Research Core, led by a PhD-level psychologist. Data coding and analysis was conducted by following the COREQ guidelines, an evidence-based qualitative methodology [33]. A hierarchical coding system was developed and refined using the interview guide and a preliminary review of the transcripts. Major categories included (1) navigator characteristics; (2) attitudes and beliefs; (3) client characteristics; (4) COVID-19 pandemic factors; (5) organizational characteristics; (6) navigation services; (7) communication; (8) injectable PrEP; (9) emotions; (10) needs and suggestions; (11) barriers and facilitators; and (12) notable quotes. Major categories were further divided from one to 10 subcategories, with some subcategories having additional levels of hierarchical division. Definitions and rules were written for the coding categories.

Experienced qualitative coders first established reliability in using the coding system on one transcript. Coding was compared and discrepancies resolved through reconciliation. Coders then independently coded the remaining six transcripts. Each speaking turn was treated as a separate quote and could be assigned up to 10 different codes. The coded transcripts were combined and sorted by code, creating an analytic spreadsheet. Transcripts, quotations, and codes were managed using Microsoft Excel 2016 (Microsoft Corporation, Redmond, WA, USA) and SPSS version 28.0 (IBM Corp, Armonk, NY, USA).

**Figure 1:**
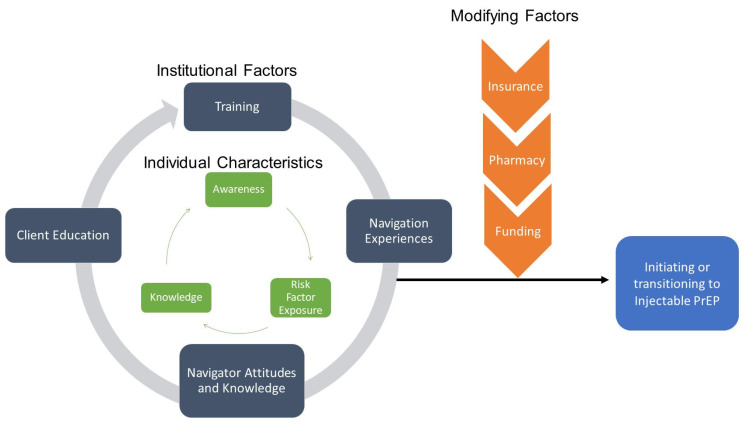
Conceptual model of characteristics and factors impacting initiation and transition to LAI PrEP.

We used an iterative inductive/deductive approach to the qualitative data analysis [33,34,35], resulting in the development of a conceptual framework (Figure 1) based on the Consolidated Framework for Implementation Research (CFIR) [36,37]. Deductively, we used knowledge of healthcare systems and inductively, we used the coded quotes to identify higher order themes and relationships between themes. The process was iterative in that the framework is theoretically informed, while the specific content in the framework is derived from the qualitative data.

## 3. Results

These results discuss each element of the conceptual framework and illustrate the discussion with quotes from navigators. At the institutional level, themes emerged that centered training of navigators, experiences navigating clients to PrEP, attitudes of navigators about LAI PrEP, and the ways in which navigators approach client education. At the individual level, themes emerged of low awareness of PrEP among key populations, misconceptions about the risk factors that increased exposure to HIV, and knowledge about how to access LAI PrEP. Using the CFIR framework, the modifying factors were barriers associated with insurance policies, availability or PrEP at pharmacies, and the centrality of funding for PrEP medications and associated medical costs.

### 3.1. Institutional Factors

Institutional factors refer to elements within the work settings of the navigators that impact the success of transitioning clients to injectable PrEP. Navigators emphasized the importance of navigator training, patient education, and navigators’ attitudes to promote effective implementation of injectable PrEP services. Navigators also shared their personal navigation experiences regarding injectable PrEP and shared what can be improved within institutions to facilitate high quality services.

#### 3.1.1. Training

Navigators discussed the training they received regarding injectable PrEP. Very few navigators reported receiving adequate training while most navigators shared that they received limited or no training. Participants indicated that this lack of training was a significant barrier towards navigating clients to injectable PrEP. Information they would want to learn more about and suggestions regarding what should be included in training were provided.

##### Training Received

The minority of participants who did receive injectable PrEP training shared their experiences. This participant shared positive experiences and noted the benefits of organized, consistent trainings:

*There were pretty consistent trainings coming out of the HIV prevention group, …. helping with all sorts of topics that they were pretty responsive to what training needs we have. …It felt very organized on their end. I felt really well connected to the other navigators, and the special project director for PrEP was very giving of her time.*

[Participant 001]

##### Limited Training

Most navigators reported receiving little or no training. This navigator shared that new navigators received training and expressed the need for experienced navigators to receive continuing education training as well:

*So, there was not any... I found out more about it through pretty much PrEP navigators, the newer PrEP navigators we had, when they went through training and stuff…I think they need for people that have been PrEP navigators for a while, people more training on it. It’s more through the grapevine is where I’m getting it from, and then I have to go do my own research on it.*

[Participant 006]

This navigator described using online sources and directing clients to providers because they have received no training:

*… I have not received any training and admittedly have not sought it out either. I just looked it up and then know to direct them to the provider with any further questions.*

[Participant 007]

These participants reported receiving limited training and emphasized the advantages of recurrent, refresher training courses on injectable PrEP to keep navigators updated with the latest guidelines:

*It’s been a while since I’ve been to the PrEP nav [igator] training. And maybe that’s kind of what they need is to do a refresher course on PrEP for the injectable. … It’s pretty much, you have to call the different physicians and ask what their guidelines are, what they’re going to do on it.*

[Participant 006]

*No, no training or preparation. There’s the PrEP navigation training that we received through TDH, and I will say, with all honesty, I wish it was something like CPR classes where you either didn’t have to go through the full thing again, but got to go through a shortened version, I think would be really helpful, especially when it comes to updates like this, because I am definitely a PrEP expert in many ways, but it would really help if there was something to facilitate or teach me and guide me to be a PrEP expert on Apretude^®^ as well.*

[Participant 007]

##### Training Suggestions

Participants touched on training uncertainties that plague the navigator process for injectable PrEP, providing insightful suggestions to enhance training and even outlined an ideal training model. Being a relatively recent development, navigators emphasized the need for experienced navigators to stay up to date through training refreshers:

*The new navigators are getting it as part of their training, but the older ones, they’re hardly getting anything from it.*

[Participant 006]

One participant outlined essential questions that injectable PrEP navigators should be equipped to answer through training:

*Now, how does an injectable version provide you with that [protection] for two months? How is this working in your system to where it’s not something that’s introduced to your body every day, to where it’s lasting that long? What are the differences in efficacy? …Have they been approved for the injectable? I Who is eligible for this? Who isn’t? Can I help out in the prior authorization process? What can I tell a client they might have to go through in order to get on it? All of those things I would want to know in the training.*

[Participant 007]

Another participant highlighted the specific need for navigators to familiarize themselves with the insurance and cost-related aspects of injectable PrEP:

*The person has to know how insurance works. They have to know about deductibles, they have to know about copays. They have to know about provider visits, the cost of that. They have to know about fee scales. If there’s an FQHC, they’d have to know what FQHC means: Federally Qualified Healthcare Centers. They have to know how pharmacies work and how they process. There’s a lot of information PrEP navigators need to know, … But what injectable PrEP has done is it’s created a little bit of confusion because pharmacies now have to purchase the drug prior to dispensing it.*

[Participant 005]

Responses demonstrate a lack of navigator training for injectable PrEP. Consistent training to be given to navigators of all experience levels, and for training to cover topics specifically associated with injectable PrEP. navigators would benefit from training that focuses on insurance information and costs of medication.

#### 3.1.2. Navigation Experiences

Navigators shared their experiences navigating clients to injectable PrEP and outlined the barriers and facilitators to the navigation processes, communication, and education with patients. The navigators also discussed their beliefs and attitudes towards injectable PrEP.

##### Navigation Protocols and Processes

Navigators discussed their experiences managing the protocols and processes associated with injectable PrEP. Responses included mixed opinions regarding the ease of navigation. This navigator describes having positive, easy experiences navigating clients to injectable PrEP:

*I’ve had two people get on it and they’ve had fantastic experiences being on it, and it was a very easy process to do. They don’t have to worry about taking the medication daily. Pretty much your job as a PrEP navigator after that is just reminding them about appointments, and making sure that they’re not having the side effects.*

[Participant 006]

This navigator highlights the importance of communicating updated information and access requirements for injectable PrEP navigation services:

*But when I’m talking to people in the field about it and stuff, and then I set them up an appointment, they said, “I can’t take it,” or, “I got to take oral first,” It’s different from what I hear from TDH [Tennessee Department of Health]. So, I don’t know if the physicians aren’t on the same page or TDH isn’t on the same page, but there’s some breakdown right there with what the rollout should be.*

[Participant 006]

*I’ve heard some people say that you have oral at first 30 days before you can do injectable. I’ve had some say that you can just go straight to injectable. I’ve heard some people say that injectable is only for men who have sex with men. I’ve heard some people say that people who inject drugs should not be using injectable at all. It’s just not been proven to be effective for them. So, I’m getting a lot of different information, and no unifying voice from TDH.*

[Participant 006]

This navigator went on to emphasize the need for navigation protocols to be modified to accommodate injectable PrEP:

*And even the navigation and PrEP nav is set up for oral PrEP. But you’re supposed to call every month to check to make sure they’re taking their medication. Doing injectable, we need to be changing the conversation, or maybe the frequency of those conversations. … as far as like how those conversations look, how often are we calling for people that are getting injectable PrEP versus oral PrEP?*

[Participant 006]

#### 3.1.3. Navigator Attitudes & Knowledge

Navigators shared their personal attitudes and beliefs about injectable PrEP services. Navigators also discussed how confident they feel with coordinating services and conveyed mixed levels of confidence regarding their ability to administer injectable PrEP services. This participant describes feeling confident and prepared to deliver injectable navigation services:

*I felt very prepared to do it. So, [county] had 17 or 18 clinics. One of those clinics was in [city], and [redacted] Hospital was one of the study sites of injectable PrEP. And so, when those patients were coming off of injectable PrEP via the study, they had to be transitioned into primary care elsewhere. And so, we were one of the organizations absorbing those patients. And so, I knew a lot about it. I mean, knew about the lab work up that was needed, what the visit cadence was, I knew about payment assistance options.*

[Participant 001]

However, most navigators reported feeling unprepared to deliver services and emphasized needing more information about aspects of injectable PrEP:

*For myself, I don’t think that I would be the most thorough advocate for that just that’s the basis of what I know. I pretty much know the surface information of, Apretude^®^, but I do think that it’s great for those clients who do not like taking pills… However, if I just had some more information, I definitely would be including that in my everyday PrEP talks to people.*

[Participant 007]

#### 3.1.4. Client Education

Navigators described how they communicate and educate clients about injectable PrEP. Navigators focused on client-centered communication and outreach efforts used to promote education. This participant recommends the use of tangible materials such as flyers to promote client education and emphasizes the importance of tailoring educational conversations to the specific needs of each client:

*I would recommend, so the injectable PrEP Apretude^®^ is developed right now by ViiV. They have materials out there; they have programs and materials and flyers about their injectable PrEP. And I would recommend that PrEP navigators get ahold of that information so that they can hand it out to potential people that are interested in injectable PrEP. … I like to cater it to the individual, listen to everything that they have to discuss so that we can navigate in the best possible way. But also, their questions are very … they’re trying to make a good decision for them about whether or not to do oral PrEP or injectable PrEP.*

[Participant 005]

According to this navigator, clients often learn about injectable PrEP through television or social media:

*So, people that are on oral PrEP are learning about injectable PrEP through television commercials or through social media, and they’re the ones that typically come to me and say, “Hey, I’m on oral PrEP. What do you think about injectable PrEP?” Or they may go to their provider on their three-month visit and say, “Hey, I’ve heard about injectable PrEP. Could that be right for me?”*

[Participant 005]

Testimonials from individuals who have used injectable PrEP would help promote community awareness and education, as suggested by this navigator:

*I would say that just more information on PrEP that comes from actual clients that are on PrEP. I feel like a lot of the times clients may just feel like this is our job to give them this information, to say all the great things about PrEP, but to actually hear real testimonials from people, I feel like that would definitely help the community and put them at ease with any preconceived notions they may have.*

[Participant 004]

Navigators discussed the education outreach efforts of their institutions. These participants report that their outreach efforts were stunted due to COVID-19, which limited the amount of people they were able to engage with PrEP:

*Yeah. Now that I’m thinking outreach efforts were probably definitely the hardest hit, I would say the people who were interested in PrEP still had some sort of way to get to it. … And so, we have to do a lot of the footwork to get it in front of people. And so, when those efforts paused, it was really only the people who already had interest and means to engage in PrEP.*

[Participant 001]

*… Essentially once a week we’re out in the community doing testing, and then we also go, we call it Going Under the Bridge, but it’s a homeless- … camp on Tuesdays. At least once a week every week we are out in the community doing testing and talking to people about HIV and HIV prevention and PrEP. But up until probably September or October of 2021, we weren’t allowed to go out and do outreach testing, which is where most of our PrEP navigation historically has come from. It wasn’t just the height of the pandemic that affected us. It was well into 2021 that it did as well because we weren’t able to be out in the community making sure that our presence was felt.*

[Participant 002]

Participants shared questions they are frequently asked by patients about injectable PrEP and reported that the most common questions they get from clients are about side effects and effectiveness of injectable PrEP compared to oral PrEP:

*They want to know if it’s better or if they want to know whether it works more effectively than the oral PrEP. That’s the first question. … And then the second question is side effects is what side effects can I expect.*

[Participant 005]

Despite navigator support and enthusiasm for injectable PrEP, many have experienced barriers associated with the delivery of navigation services for this medication. These issues involved the protocols and processes of navigation, lack of confidence providing navigation services, and pauses on education outreach efforts. Navigators need education to know how to manage injectable PrEP protocols, meet the needs of their clients, and build confidence in their ability to administer services. Facilitation of community outreach efforts is also needed to engage individuals with this medication.

### 3.2. Individual Characteristics

Individual characteristics refer to attitudes, knowledge, and attributes of clients that impact the initiation or transition to injectable PrEP. Navigators discussed patients’ attitudes and knowledge about injectable PrEP and highlighted how injectable PrEP can benefit clients who may be exposed to specific risk factors of HIV.

#### 3.2.1. Knowledge and Awareness

Navigators discussed clients’ knowledge and awareness of injectable PrEP and the associated risks of HIV infection. This participant commented on the general need for heightened PrEP awareness, prior to HIV diagnoses:

*… but for our homeless patients and for our patients in the substance use treatment clinic, just asking them, what is your awareness of PrEP? … do you just need education specific to HIV and substance use? Because so much of it in respect to PrEP is usually around MSM. So that’s the information I wish we had, it’s just from a program improvement perspective, what do these special populations want? Or what is their actual awareness of it? … we’ve talked to homeless people and a lot of them are either, they’re only aware of PrEP if they have someone in direct contact with them has HIV, or they found out about PrEP after they’d already been given an HIV diagnosis. So, I think just what would messaging for them look like?*

[Participant 001]

One navigator noted how many individuals have limited knowledge about their susceptibility to HIV:

*... for HIV and so it’s okay to have a conversation with someone about how they are in fact at risk and that they have options to protect themselves, specifically individuals who inject drugs because historically everyone’s been taught that HIV is a gay disease and so the reality is that people truly just don’t know that they’re at risk even though they are, and that it’s okay to have that conversation with people.*

[Participant 002]

#### 3.2.2. Risk Factor Exposure

Navigators described the benefits of injectable PrEP, particularly for high-risk populations. High risk populations include people who inject drugs (PWID), engaged in sexual relationships with partners who have HIV, and/or experiencing housing insecurity. Some navigators emphasized the importance of injectable PrEP, rather than oral PrEP, for individuals injecting drugs:

*So, we have a syringe exchange program at our clinic. So, we do needle exchange for injection drug use. We identify lots of people that are injecting drugs to be candidates for PrEP. And people have accepted PrEP offers. Oral medication is not typically good for people who inject drugs because adherence is the issue. But if they’re coming in to exchange needles on a regular basis, then they can come in for their injection, for their Apretude^®^ injection.*

[Participant 005]

*People that can’t take medication daily, people who inject drugs is a good example. It’s been pushed really heavily in that population. But as far as what I’ve heard from Gilead and stuff, Descovy^®^ has not been proven to work. People who inject drugs, there has not been a study on that specifically, but it’s still been really hard in that population. Because there’s no indicators that it shouldn’t work. But that population isn’t very good at taking PrEP daily.*

[Participant 006]

Other navigators illustrated how injectable PrEP dramatically could help those who are unhoused:

*… we try to navigate to PrEP that are experiencing homelessness. Long lasting injectable PrEP would be not only life changing for that specific population, but very potentially lifesaving because the reality is that for a lot of reasons when someone is experiencing homelessness, it’s very easy to lose your medication. … Or to have it stolen because it’s in your bag, but also, you’re homeless and so there’s a lot of competing priorities every day as far as making sure that you survive to the next.*

[Participant 002]

*… we were excited when we were hearing about injectable PrEP because we were like, oh, this is great because this is our silver bullet for our homeless patients who we can’t get to take a daily oral med. …And then we find out that we can’t put these patients on injectable PrEP unless they have some sort of high-risk sexual behavior code. So, if their primary risk for HIV was IV drug use, we can’t put them on-...They’re not eligible.*

[Participant 001]

### 3.3. Modifying Factors: Policy

Modifying factors refer to external elements outside the navigators’ control that affect the outcomes of injectable PrEP services. Health insurance policies, pharmacy policies and protocols, and the costs associated with injectable PrEP are critical barriers affecting the transition to injectable PrEP.

#### 3.3.1. Health Insurance

Health insurance policies can create challenges associated with the accessibility of injectable PrEP. This navigator explains that very few of their clients have received injectable PrEP due to rejection by insurance companies:

*… most of the time that’s just an issue of insurance. We don’t see a lot of insurance companies wanting to pay for that. It’s a pretty extensive process with prior authorizations and everything. Insurance companies want to pay for generic Truvada^®^, they don’t want to pay for name brand Truvada^®^ or Descovy^®^, let alone the injectable. And then we haven’t seen any uninsured clients get injectable PrEP.*

[Participant 007]

Navigators described barriers created by insurance policies that require clients to try cheaper drugs before trying injectable PrEP.

*… But the other thing with insurance companies is that even if you’re sending in those prior authorizations and everything, they want people to try the cheapest method first. So, it would be very difficult if somebody’s coming in for the first time to get them on injectable PrEP without trying first generic Truvada… But if somebody wants an injectable, then the issue isn’t necessarily like, “No, they want the injectable. They don’t want to take a pill every day.”*

[Participant 007]

*… most insurance is going to go, well, ‘why would we pay for an expensive injectable formulation when a much, much cheaper oral formulation is available?’ … And so, it was one of these weird catch 22s of like, it was most accessible for patients without insurance. Because then you could do the manufacturer PAP [Patient Assistance Program].*

[Participant 001]

This navigator explains that insurance company policies limit access to injectable PrEP by denying coverage for clients who want to use it for convenience:

*“Okay, well, I want the injectable, but I have to try this.” And then it’s like, okay, well, then the question becomes, is it causing nausea? Is there another reason that you want the injectable? And it doesn’t seem to be a good enough reason to insurance companies that people just want it for convenience. That’s the whole reason, so it’s really hard to make that argument if that’s the standard that they’re having for paying for it.*

[Participant 007]

This navigator shares that some insurance companies do not adhere to policies that help clients obtain injectable PrEP:

*But policies are already there, that’s my point. But the insurance companies aren’t practicing that… They’re saying, “No, we want you to take oral PrEP instead. We don’t want to pay for the injectable PrEP; it’s too expensive.” But the policy is there. ….….…. That’s the barrier right there.*

[Participant 005]

Another navigator relayed their frustration towards insurance companies that refuse to cover injectable PrEP:

*… why waste all this money making something just for insurance companies not to cover it? … But also, as an insurance company or an insurance plan, why would you not want to cover it if it’s going to prevent people from getting HIV because if they get HIV, you don’t have a choice, you have to cover their HIV medication, and it is expensive…*

[Participant 002]

#### 3.3.2. Pharmacy

Pharmacy protocols and policies can prevent clients from receiving injectable PrEP and/or cause delays in receiving it. This navigator described delays associated with the timing when pharmacies have to order injectable PrEP:

*… We issue oral cabotegravir, which the oral version of Apretude^®^ for 30 days so that we can experience whether or not the client is experiencing side effects or there’s issues with insurance. So, we get that sort of on the front end sorted out with insurance so that they’re not waiting for an injection, that they’re not waiting for the pharmacy to get it in. They’re not waiting for the pharmacy to swipe the card because the pharmacy, remember, has to purchase it ahead of time. So, there may be three or four days after the provider has written an Apretude^®^ script before the client realizes that their insurance isn’t going to cover it. So now they got to go back to the provider to get a new script.*

[Participant 005]

*… one of the barriers is the pharmacy having to purchase ahead of time or to order it after the prescription comes in, because some pharmacies will wait to order. Once they get a prescription, they’ll order it and they’ll swipe the card to see if insurance will pay for it. So, they know upfront whether it’s going to be accepted or not before they order it. But that’s a four-day delay because it’s going to take four days if the drug is available to be shipped to them.*

[Participant 005]

These quotes from a participant describe logistical barriers associated with pharmacies retrieving and mailing injectable PrEP:

*I would say that our frustration within all the logistical burden, especially with pharmacy, to actually get it, if a patient came to me and said they were interested in being on injectable PrEP, I’m pretty confident, and I could have given them a summary of what it would take to get them on it. But it was just such a cumbersome process compared to oral PrEP…*

[Participant 001]

*… we needed training on was things like prior authorizations, and pharmacy navigation of what pharmacies, what does it mean to get this at a pharmacy in your area? Which pharmacies are going to have it? And just the logistics of getting it mailed. Because there were all sorts of stuff about, it’ll be mailed to you frozen and it has to thaw and all this stuff.*

[Participant 001]

#### 3.3.3. Cost/Funding

The cost of injectable PrEP creates barriers that make the medication difficult to obtain for clients. This navigator voices the need for information regarding how to get injectable PrEP paid for through funding:

*Just more information about more ways to get it paid for. We’re very heavily dependent on using 340B here. So, I know with the oral PrEP, we’ve went over like Gilead Copay Assistance Program, Patient Assistance Program, and then GoodRx. I think they do the same thing with injectable… If we did an interview type situation where we went through a mock navigation call for oral PrEP, I need to do that for injectable PrEP too. The conversation’s going to look different. Whereas oral PrEP price and how many times they take PrEP a week, kind of go over the percentage of the efficacy with it. With injectable PrEP the conversation may be totally different. “Hey, you need your appointment by this day…”*

[Participant 006]

This navigator indicated that their institution may no longer be able to help clients cover copays for injectable PrEP due to funding legislation changes in the state of Tennessee:

*…We’re among them, to where we might not be able to cover the copay anymore, or the injectable unless we find a different option. We just won’t have the money to do it. So come June, July, (2023) you may see a very different conversation about how difficult it is…it may be a very different experience come June, July when this funding change happens. Are people going to be able to get that paid for anymore?*

[Participant 006]

Questions and concerns about cost of injectable PrEP are often raised by clients, according to this navigator:

*Like where is it available and are there any side effects and is the cost any different? That’s the main one that I’ve gotten, is that is the cost any different.*

[Participant 004]

This navigator indicated that cost barriers can lead to dangerous delays and jeopardize ongoing injectable PrEP administration:

*Yeah, it’s a cost issue.... Because the oral PrEP is for three months, and injectable PrEP is for two months, if there’s delays in your appointment, if there’s delays in the pharmacy getting the medication in, that can mean that there’s a lapse in your treatment. And that lapse in treatment can lead to exposure to HIV. So, I would like to see that smoothed out.*

[Participant 005]

One navigator raised concerns over cost resources that were lost during the pandemic and explains how this loss can keep institutions from meeting PrEP medication needs:

*I think one of the first things is just the lack of practical support resources that we lost during the pandemic. As far as patients needing transportation to and from appointments to get their three-month lab work done, that typically became an issue. Then whenever patients were having insurance options or billing options, there was just no way that they can be able to get those needs met… to continue take their PrEP medications and things of that nature.*

[Participant 003]

Modifying factors such as insurance policies, pharmacy protocols, and costs associated with injectable PrEP have limited clients’ access to the medication and have restricted institutions’ abilities to meet their needs. Policy and protocol changes within insurance companies and pharmacies were advocated to promote accessibility of PrEP. Helping clients afford costs associated with injectable PrEP was also a major concern. Navigators need support and information regarding how to address these barriers in order to help clients initiate or transition to injectable PrEP.

## 4. Discussion

While PrEP has been available for more than a decade, a lag in uptake in southern US states like Tennessee among communities of color and PWID has been observed [3,22]. PrEP navigation has been used to improve the pace of PrEP uptake and must integrate new forms of PrEP as they are approved. The development of a compendium of PrEP modalities will allow for multiple populations of focus to choose which version of PrEP suits their needs [38]. This study of state health department sponsored PrEP navigators used interviews to understand institutional-level factors, individual-level factors, and modifying factors of implementing LAI PrEP in Tennessee. This study will contribute to the dissemination of LAI PrEP among populations most affected by HIV. LAI PrEP was approved at the end of 2021, but uptake has not been robust. LAI PrEP is the first non-oral modality for HIV PrEP approved in the US [25], with the expectation that additional modalities will be introduced in coming years [39]. Clearly delineating challenges to dissemination and initiation of approved PrEP modalities will greatly influence the impact of new products as they are introduced.

Navigators reported that training varied greatly at the institutional level, with only a minority receiving training specific to LAI PrEP. LAI PrEP knowledge was integrated into the training of newer navigators, but more established navigators would need an updated training. The training gap diminished navigators’ readiness to introduce clients to LAI PrEP. Updated training would also help when there are discrepancies in how LAI PrEP is prescribed, in this case, making optional the oral-pill lead-in used in the LAI PrEP and HIV treatment approval studies [24,40]. In addition to more training for established navigators, trainings should include estimating costs, and facilitating navigators’ ability to identify and engage with resources for accessing and paying for LAI PrEP. Better trained navigators would be more likely to feel prepared and confident to navigate clients to LAI PrEP.

At the individual level, navigators said that potential clients maintained the perception that PrEP eligibility was based solely on sexual risk and targeted only for gay, bisexual, or other men who have sex with men, which is consistent with the previous literature [41,42]. Groups such as heterosexuals, those experiencing homelessness, and PWID were not as aware of PrEP or had little knowledge of PrEP access, as has been noted previously [2,43]. While the most recent CDC guidelines [44] and US Preventative Service Taskforce recommendations [45] are much broader than before, this may not have been disseminated to all populations who could benefit from PrEP. Among PWID, a study in Maryland found that PrEP barriers included cost, misperceptions about who was a candidate for PrEP, and stigma [46]. Studies to specifically understand LAI PrEP use among PWID and those experiencing homelessness are being proposed now, but such dissemination will also require greater understanding of factors relating to access and affordability, and ultimately better training for navigators and clinics that serve these populations.

Three interrelated modifying factors were identified for initiating and transitioning to LAI PrEP. Given its relatively recent approval, a lag in insurance awareness and willingness to cover LAI PrEP versus pill-based regimens was highlighted. In some cases, insurance companies were reticent to initiate clients on LAI PrEP unless they had first attempted, or in some cases, been adherent to, pill-based regimens. Relatedly, delays occur in clients receiving LAI PrEP as pharmacies may wait to order the LAI PrEP formulation until after acceptance by the insurance company is confirmed. Finally, cost of LAI PrEP, regardless of insurance coverage, remained a barrier to uptake. The varying nature of available programs to assist with PrEP costs requires navigators and clients to consistently monitor program changes and policy. Although navigators suggested mock LAI PrEP assistance calls as a part of future PrEP navigator training, there remains concern about how marginalized people and populations with limited resources will maintain access to LAI PrEP, a stark reminder of how the social determinants of health influence HIV rates in the US South [43,47].

These interrelated modifying factors are consistent with previous literature specifying barriers to PrEP uptake, particularly in the US South: namely insurance coverage and the cost of LAI PrEP [32,48]. Nationally, three programs from Gilead Sciences, ViiV Healthcare, and GalaxoSmithKline/Pfizer provide support for PrEP cost-sharing. Despite calls for a national HIV PrEP program to finance PrEP, no such program yet exists [49]. Advocates of a national program note that federal bulk purchasing of PrEP products may lower the costs for those least likely to have PrEP access, including those uninsured, while increasing laboratory access and locations for PrEP access [49,50]. There are some states who have instituted their own PrEP cost sharing programs, of which Tennessee participates with pill-based forms of PrEP in its formulary [48], but does not currently include LAI PrEP [51]. Several studies have shown greater PrEP uptake in states with Medicaid expansion [52,53], and this suggests that perhaps the most promising policy recommendation in support of increasing PrEP uptake is the expansion of Medicaid in Tennessee [32,52]. Another policy consideration is the use of hub-and-spoke versions of PrEP provision- a form of PrEP prevision that allows an attending nurse or other clinician to complete the clinical assessment while connected virtually to a prescribing clinician [32]. A policy change allowing for this type of in-office alternative may be best utilized in proving HIV prevention care to people experiencing homelessness but regularly attend a syringe service program (SSP) or other periodic service offered by PrEP navigators and their clinics. Taken together, these policy considerations at the national, state, and clinic levels may be used to increase PrEP uptake and expand access to additional populations.

There are several limitations to this study. The sample size for this study included seven navigators from across the state of Tennessee, however most themes were consistent across participants. During the study period, substantial changes were announced for Tennessee that may have influenced participation in the study. In January 2023, under direction of the commissioner of the Tennessee Department of Health, the state announced that it would no longer accept federal funds from the Centers for Disease Control and Prevention to support HIV testing, surveillance, and HIV prevention services nor would the state accept funding for Ending the HIV Epidemic efforts; instead the state would “assume direct financial and managerial responsibility for these services” with an effective date of June 2023 [54]. Tennessee is the only state to refuse federal funds for HIV prevention, concerning many across the state including policy experts [55]. Such a policy shift may have had a chill effect on would-be participants, as the study was focused on PrEP navigators funded through the state health department. The exact locations (i.e., organization, county) and demographic information were not included for participant privacy given the small sample size; this represents several limitations in that any differences in perception by gender or racial/ethnic background could not be identified. In addition, PrEP navigators in rural areas were underrepresented compared to urban navigators, which is an important limitation to generalizability of findings and a consideration for scaling PrEP across the state. This sample only includes PrEP navigators supported by the state health department and does not represent individuals who perform navigation tasks at other clinics or providers (e.g., primary care providers, clinics who are separately funded). Lastly, this analysis focuses on the perceptions and experiences of PrEP navigators, only one piece of a complex system of individual, interpersonal, institutional, and political factors interacting within the social determinants of health.

## 5. Conclusions

Taken together, the use of the CFIR framework helps to clarify multiple pathways to successfully scaling new PrEP products. Data show that (1) ensuring that all populations are aware of PrEP modalities, (2) developing tools to identify the clients for whom the new modality is most appropriate, and (3) preparing institutions such as insurance companies, pharmacies, and clinics about how to rapidly access and pay for emerging modalities, are necessary in improving efforts to end the HIV epidemic. Further, while navigators identified barriers and facilitators in their clinics, it must be noted that several states are considering expanding where the public can access PrEP, such as in pharmacies [56,57]. The rapid development of new HIV prevention tools will require researchers and practitioners alike to consistently engage all stakeholders in the implementation of established and emerging PrEP modalities if they are to be equitably scaled to end the HIV epidemic.

## Data Availability

The data that support the findings of this study are available on request from the corresponding author. The data are not publicly available due to privacy or ethical restrictions.

## Abbreviations

The following abbreviations are used in this manuscript:

PrEP	Pre-exposure prophylaxis
MSM	Men who have sex with men
PWID	People who inject drugs
NLRR	Navigation, Linkage, Reengagement and Retention
LAI	Long-acting injectable
CFIR	Consolidated Framework for Implementation Research
TDH	Tennessee Department of Health

## Appendix A

### Appendix A.1. Qualitative Interview Guide Questions

Note: Questions for this analysis have been taken from a longer interview guide. Below are the specific questions used to understand PrEP navigator perceptions for initiating and transitioning clients to LAI PrEP in Tennessee.

**Introduction:**

Thank you for agreeing to be a part of the study. We will begin by asking a few questions about your role as a PrEP navigator.

(1) Please tell me about your role as a PrEP navigator at your organization. Please include how long you have been in the role (*approximations are fine*).
  - Clarify, prompt: Can you share a bit about your day-to-day activities as a PrEP navigator?

(2) Please describe the population for which you provide PrEP navigation.
(3) Thank you for your responses so far. I would now like to discuss long-acting injectable PrEP. Can you describe how prepared you feel to navigate potential PrEP users to injectable PrEP, also known as Apretude^®^?
  (a) Can you tell me a bit about your experience in navigating clients to injectable PrEP?
  (b) Can you share what kinds of questions clients have asked when discussing injectable PrEP?

(4) Can you describe any training or preparation that you have received in terms of transitioning current PrEP users to long-acting injectable PrEP?
(5) If you could design a training for PrEP navigation, including injectable PrEP, what would that training include?

**Closing Question:**

(6) Thank you for sharing your experiences of PrEP navigation with me. Are there any additional thoughts that you would like to share?
